# Trans-obturator cable fixation of open book pelvic injuries

**DOI:** 10.1038/s41598-021-92755-2

**Published:** 2021-06-29

**Authors:** Martin C. Jordan, Veronika Jäckle, Sebastian Scheidt, Fabian Gilbert, Stefanie Hölscher-Doht, Süleyman Ergün, Rainer H. Meffert, Timo M. Heintel

**Affiliations:** 1 Department of Orthopaedic Traumatology, University Hospital Würzburg, Julius-Maximilians-University Würzburg, Oberdürrbacher Str. 6, 97080 Würzburg, Germany; 2 Department of Orthopaedic Surgery, University Hospital Bonn, Venusberg-Campus 1, 53127 Bonn, Germany; 3 Department of Orthopedics, Physical Medicine and Rehabilitation, University Hospital LMU München, Marchioninistr. 15, 81377 München, Germany; 4 Institute of Anatomy, Julius-Maximilians-University Würzburg, Koellikerstraße 6, 97070 Würzburg, Germany

**Keywords:** Anatomy, Medical research

## Abstract

Operative treatment of ruptured pubic symphysis by plating is often accompanied by complications. Trans-obturator cable fixation might be a more reliable technique; however, have not yet been tested for stabilization of ruptured pubic symphysis. This study compares symphyseal trans-obturator cable fixation versus plating through biomechanical testing and evaluates safety in a cadaver experiment. APC type II injuries were generated in synthetic pelvic models and subsequently separated into three different groups. The anterior pelvic ring was fixed using a four-hole steel plate in *Group A*, a stainless steel cable in *Group B*, and a titan band in *Group C*. Biomechanical testing was conducted by a single-leg-stance model using a material testing machine under physiological load levels. A cadaver study was carried out to analyze the trans-obturator surgical approach. Peak-to-peak displacement, total displacement, plastic deformation and stiffness revealed a tendency for higher stability for trans-obturator cable/band fixation but no statistical difference to plating was detected. The cadaver study revealed a safe zone for cable passage with sufficient distance to the obturator canal. Trans-obturator cable fixation has the potential to become an alternative for symphyseal fixation with less complications.

## Introduction

Disruption of the pubic symphysis is commonly seen in pelvic ring injuries of trauma patients^1,2^. The disruption of the anterior pelvic ring might occur in combination with a posterior pelvic ring impairment of variable severity. When diastasis of the disrupted symphysis pubis exceeds a certain displacement, stabilization of the anterior pelvic ring is recommended^3,4^. Adequate reduction and stable fixation can restore pelvic ring alignment and allow early patient mobilization^5^. Currently, symphyseal plating represents the most common technique for anterior pelvic ring fixation in such conditions^6^. This plate fixation, however; represents a static fixation of what is actually a dynamic junction. The pubic symphysis comprises a fibrocartilaginous disc between the articular surfaces of the pubic bones, encapsulated and reinforced by surrounding ligaments allowing limited movement^7^. From the healing perspective, the currently preferred treatment is not the ideal one as it does not provide a dynamic fixation^8^ and the plate is placed in an unfavorable rectangular position to the load vectors that affect the ruptured symphysis. Other disadvantages of plating include hardware breakage or implant loosening, which lead to recurrent instability of the pubic diastasis. These implant failures are the main reasons for revision surgery and may be linked to the rigid character of the fixation technique^9^. More dynamic fixation techniques like a trans-obturator cable cerclage could bypass this problem but have not been taken up in daily practice. Clinical and laboratory studies on pubic wiring are inconsistent and often small in size^10–15^. Recent studies only consider simple wiring instead of a more stable cable-system fixation^16^. The aim of this study is to analyze dynamic trans-obturator cable fixation as an alternative stabilization for the disrupted pubic symphysis. The hypothesis is that, compared to plating, trans-obturator cable fixation provides an equal or even superior fixation strength. If so, the cable fixation could be a dynamic, less invasive alternative to symphyseal plating with less complications.

## Methods

### Specimen and fracture generation

Thirty synthetic pelvises (Pelvis Complete, Synbone, Art. No. 4060) were used. In this model, the sacroiliac joint and the symphysis are joined using flexible plastic foam. There are no ligaments or muscles attached to the pelvis. An anterior–posterior compression injury (Young and Burgess APC II; OTA/AO: 61-B2.3d) was simulated, in which the pubic symphysis and sacroiliac joint connection (anterior only) on one side was disrupted. Preparation and fixation of posterior instability was conducted in all specimens using the same technique. A 3.5-mm drill was used to open the cortical bone and create an osseous corridor to S1 and S2 while the pelvis was still intact. Final fixation was done with partially threaded 7.3-mm-diameter, 90-mm-long cannulated titan screws, including washers.

### Experimental groups

**Group A**, the control group, received traditional plate fixation (N = 10). Here, pubic disruption was fixed using a four-hole stainless steel symphysis plate (Symphyseal Plate 3.5 with coaxial combi-holes, DePuy Synthes; Art. No. 02.100.004) and 3.5-mm self-tapping, non-locking cortex screws. The medial and lateral screws were 55 mm and 60 mm long, respectively. Medial screws were placed with lateral inclination and lateral screws were tilted towards the symphysis^17^.

Pubic symphysis disruption in **Group B** (N = 10) was fixed with a trans-obturator cable, using a 1.7-mm-diameter interwoven stainless steel cable and cable crimp (cable with crimp, DePuy Synthes, Art. No. 298.800.01S). A medium size cable passer was used to tunnel the cable twice through the obturator foramen and embrace the pubic symphysis. The cable tensioner in combination with the provisional tensioning device and attachment bit pre-tensioned the cable wire up to 40 kg. Care was taken that the cable did not fully cut into the cortex. Final fixation was conducted with the cable crimper. The cable was shortened near the tip with an adequate cutter.

**Figure 1:**
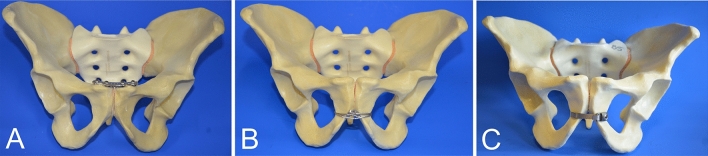
Different groups tested. (**A**) Stabilization with a four-hole 3.5-mm stainless steel plate. (**B**) Trans-obturator wire using a 1.7-mm cable system. (**C**) Trans-obturator fixation using a broad titan band. Ten specimens were tested in each group.

**Figure 2:**
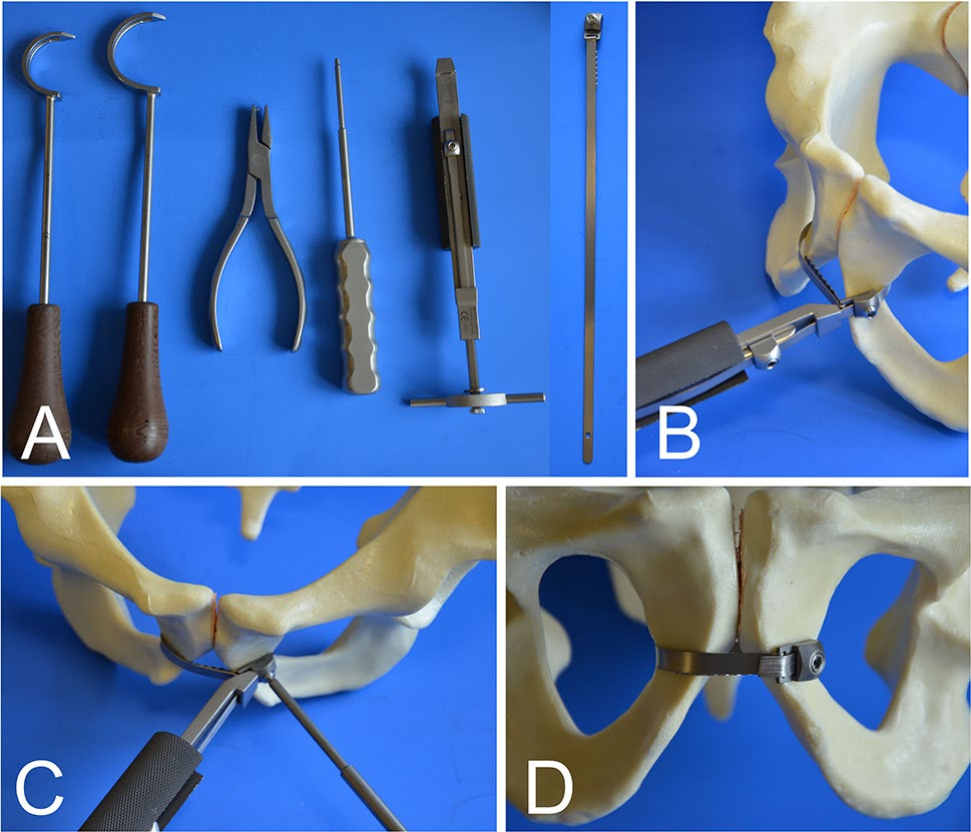
Stepwise application of a trans-obturator fixation in Group C. (**A**) Instruments and titan band (**B**) Tensioner (**C**) Fixation of the clamp using a screw driver (**B**) Final position.

A titanium cerclage band was used in **Group C** (N = 10). The cerclage band was 5.8 mm wide and 240 mm long (Titanium-Cerclage Band according to Thabe, Link, Art. No. L63-4300/02), and was used with a cerclage band guide of the appropriate size. The cerclage was passed through the obturator foramen around the pubic symphysis, ensuring broad contact of the cable band to the bone. A cable tensioner was used for reduction of the anterior pelvic ring and tightening the cable. The tensioning device did not allow measurement of the applied tension. The cable was locked using a hexagon screwdriver and shortened using a cutter (Figs. 1 and 2).

### Biomechanical test setup

**Figure 3:**
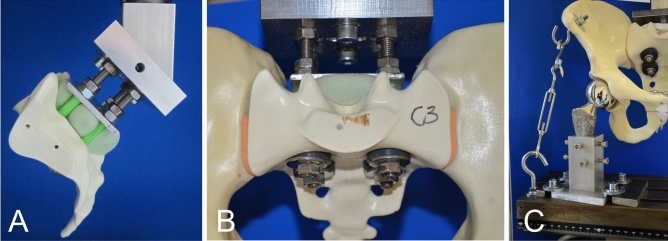
Biomechanical testing. (**A**,**B**) Fixation of the pelvis to the material testing machine. (**C**) Single-leg-stance model in which load can be applied to one side.

A single-leg-stance model was established and testing was performed using a universal testing machine (Z020; Zwick/Roell) and testXpert II software (Version 3.6; Zwick/Roell; https://www.zwickroell.com/de/zubehoer/pruefsoftware/testxpert-ii/)^18,19^. Pelvic samples were attached at the sacrum to the testing machine at a physiological 45° tilt using a custom-made aluminum device. A hemiarthroplasty prosthesis was used for articulation with the acetabulum at 15% adduction on one side to simulate unilateral axial load. The femoral stem was wedged in a steel quiver attached to the bottom of the machine. Photographic documentation of the pubic symphysis was conducted throughout the testing. In contrast to previous studies by the authors, a cable-pulley system simulated the abductor muscles to increase maximum load levels^18–20^. A series of pretests were performed to establish the protocol and summarize the data for power analysis. The pretests encompassed load levels of 50 to 1000 N and test cycles of 500 to 3000 repetitions. All pretests indicated that 200, 400, and 600 N were valuable load levels, and that test cycles of 500/1000/1500 repetitions were adequate to show differences between the experimental groups. The decision to use such load levels was based on in vivo measurements, the literature and previous work by the authors^18–23^ (Fig. 3). The main tests were started with 10 setting cycles at 0- to 10-N loads at 50 mm/min frequency. A load–displacement curve was generated during the testing. Outcome measurements were peak-to-peak displacement at 200, 400, and 600 N, total displacement, plastic deformation, and stiffness.

### Surgical approach

A cadaver study was conducted to understand the relationship of the trans-obturator cerclage to surrounding structures. Fresh frozen male and female cadavers (one of each) were placed in a supine position. Using a Pfannenstiel approach, a slightly curved 15-cm-long horizontal incision was made, centered about 2 cm cranial to the pubic symphysis. The anterior portion of the rectus sheath was prepared and the rectus sheath was divided to identify the rectus abdominis and the pyramidalis muscles. The muscle was separated from the attachment at the pubic bone. For better exposure, dissection was continued laterally to the external inguinal ring, the spermatic cord/round ligament, and the vascular and muscular lacuna. Dissection of the obturator foramen was conducted, including the obturator canal and membrane. A 1.7-mm cable wire was passed through the medial part of the obturator foramen and fixed. The distance to the surrounding anatomical structures was evaluated.

### Statistical analysis

A power analysis performed using a power of 80% and a significance level of 5% showed that the sample size was adequate. The results are presented as mean values with standard deviation. All data underwent statistical analysis for normal distribution using the Shapiro–Wilk test. Analysis of variance was used to compare the means. A p value < 0.05 was considered to indicate significance (SPSS Statistics, Version 25, IBM; https://www.ibm.com/de-de/analytics/spss-statistics-software). As no statistical differences were found, further tests were not conducted.

### Ethics declarations

This article does not contain any studies with human participants or animals performed by any of the authors.

## Results

### Peak-to-peak displacement at 200, 400, and 600 N

**Figure 4:**
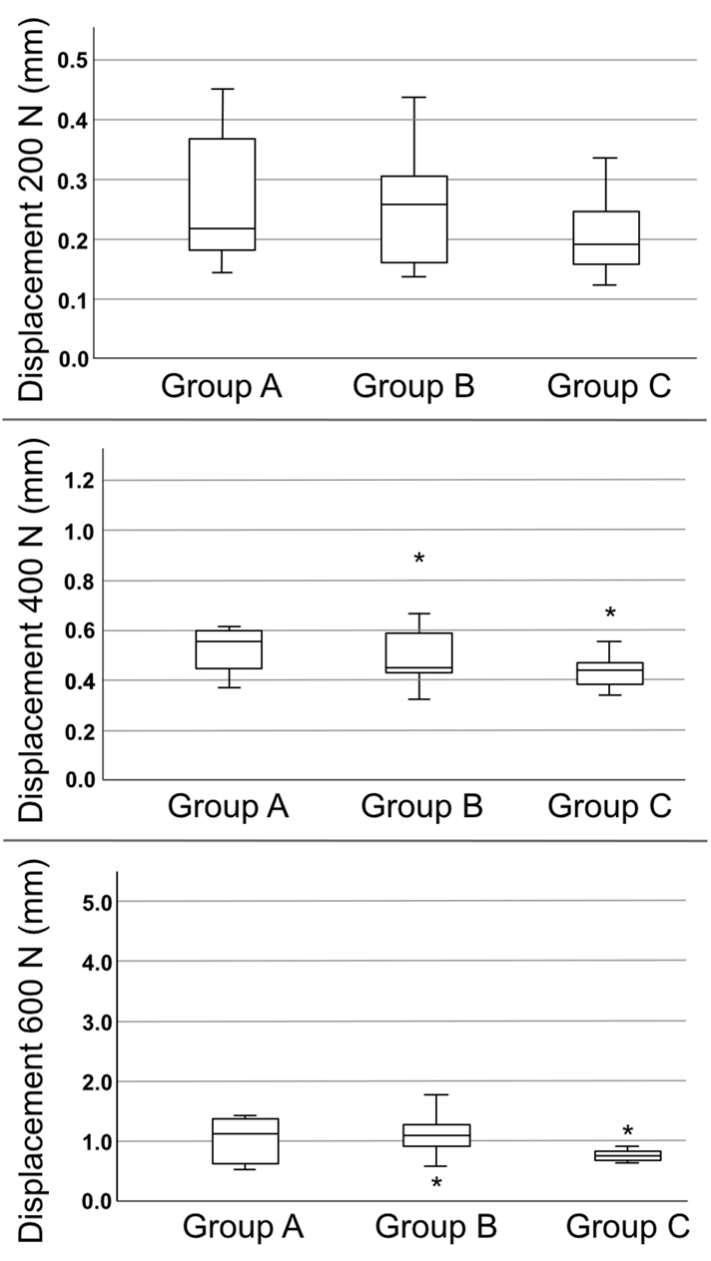
Peak-to-peak displacement of the different groups tested under 200-, 400- and 600-N Load. Displacement was measured for the whole pelvis. Displacement rises under increasing load. No implant failure was observed. Statistically significant differences were not detected between the groups (asterisk = outlier).

Peak-to-peak displacement was measured for the entire pelvic ring. Under 200 N, there was a mean peak-to-peak displacement of 0.27 ± 0.10 mm in group A, 0.24 ± 0.98 mm in group B, and 0.20 ± 0.06 in group C. Under 400 N, the mean peak-to-peak displacement was 0.57 ± 0.21 mm in group A, 0.50 ± 0.16 mm in group B, and 0.45 ± 0.09 in group C. Under a 600-N load, the values were 1.16 ± 0.71 mm in group A, 1.34 ± 1.0 in group B, and 0.78 ± 0.15 in group C. There were no significant differences between the groups (p = 0.29; p = 0.27; p = 0.25; Fig. 4).

### Total displacement

The mean total displacement was 3.96 ± 0.42 mm in Group A, 3.58 ± 0.54 mm in group B, and 3.70 ± 0.43 mm in Group C. There were no significant differences among the groups (p = 0.20).

### Plastic deformation

Plastic deformation was measured using a load–displacement curve. It represents the irreversible deformation of the whole pelvic ring at 200-N load. The mean plastic deformation was 0.63 ± 0.20 mm in group A, 0.55 ± 0.17 mm in group B, and 0.56 ± 0.19 mm in group C. There was no significant difference among the groups (p = 0.58).

### Stiffness

**Figure 5:**
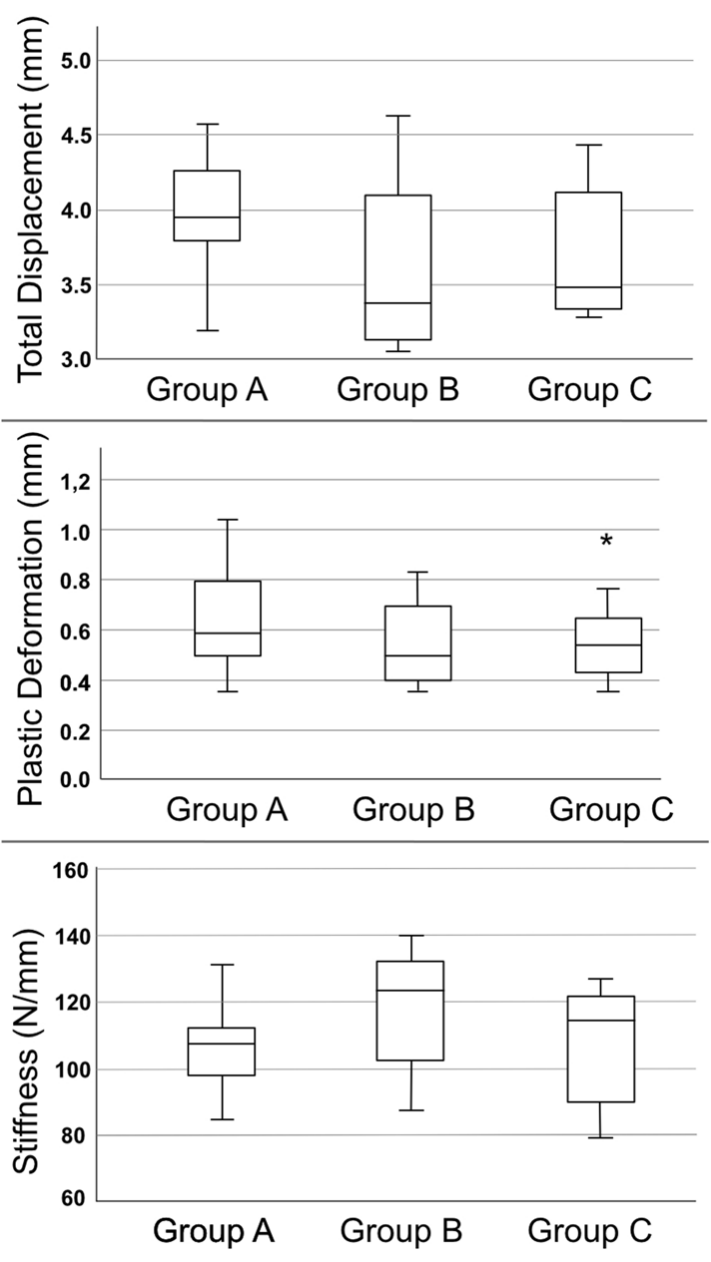
Total displacement represents the final change of the probe after all load levels were applied. Plastic deformation represents the permanent and irreversible strain of the hole construct. The plate and cable systems showed only minimal amounts of deformation. Even though group B had the highest stiffness, no statistical difference was found.

Stiffness was measured using the slope of the load–displacement curve at 400 N. The mean stiffness was 105 ± 13 N/mm in group A, 118 ± 18 N/mm in group B, and 107 ± 16 N/mm in group C. There was no significant difference among the groups (p = 0.21; Fig. 5).

### Surgical approach

**Figure 6:**
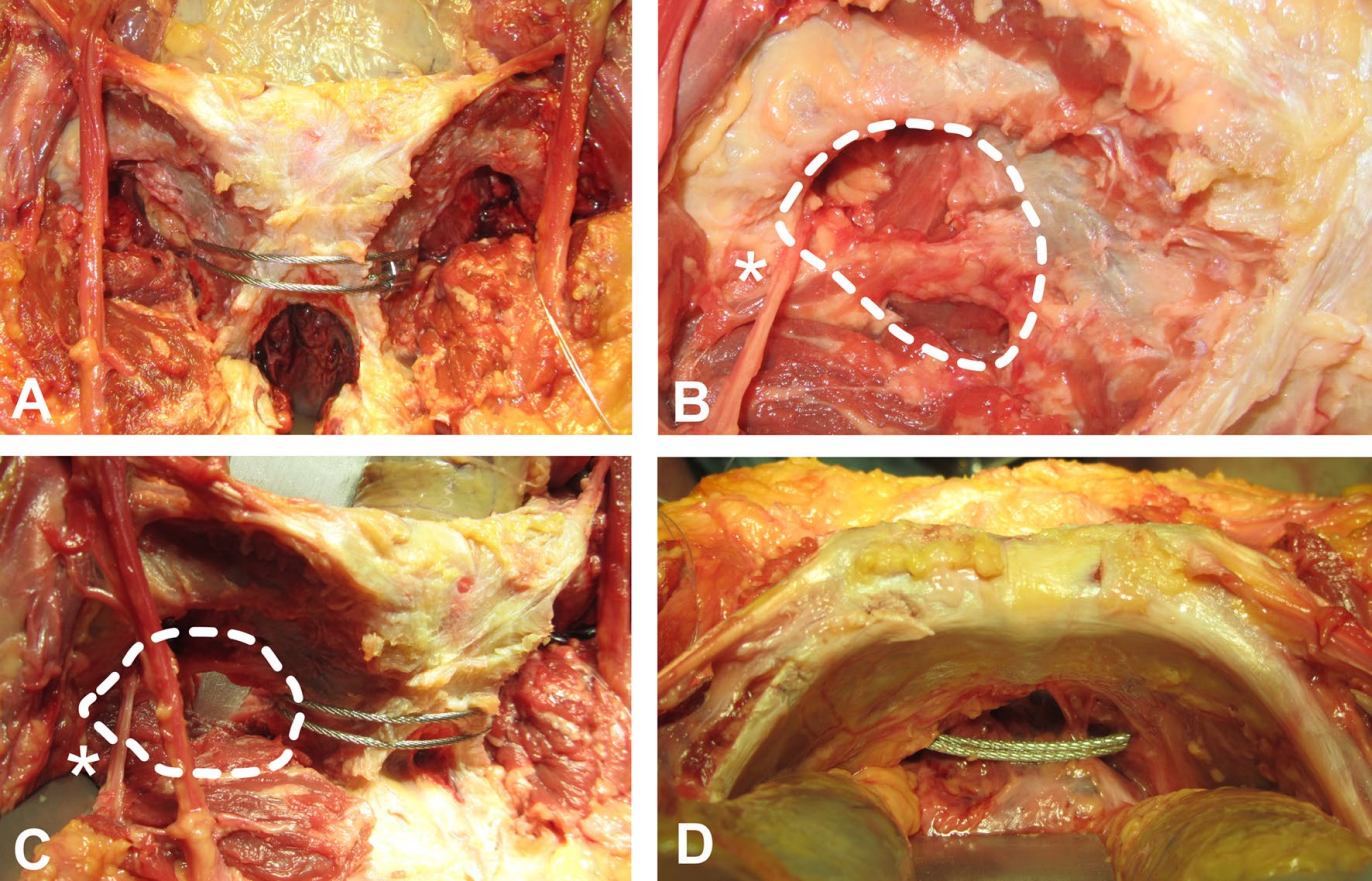
Anatomical landmarks. (**A**) Pubic symphysis anteriorly fixed with a trans-obturator cable. (**B**) Dotted line encircles the obturator foramen. The membrane does not seal the whole foramen. The asterisk marks the vessels and the nerve leaving for the obturator canal. (**C**) Obturator foramen from a more lateral perspective. Note the distance to the cable system. (**D**) View from the cranial pubic rim of the pubic branch. The bladder is retracted.

**Figure 7:**
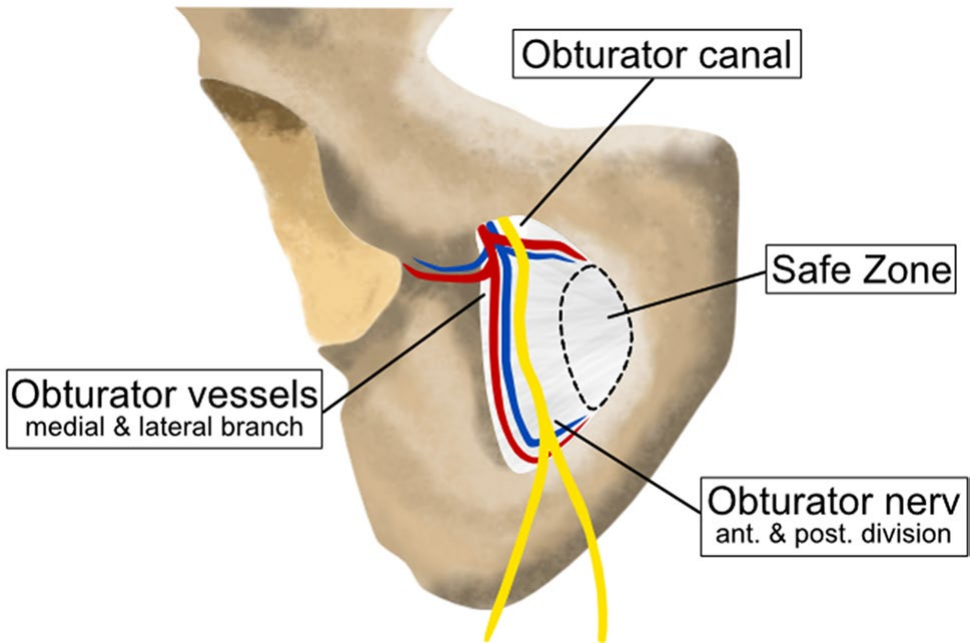
Schematic illustration of the obturator foramen. The obturator artery divides into medial and lateral branches as it emerges from the obturator canal. An acetabular branch rises from the lateral part and runs towards the hip joint. The obturator nerve divides into anterior (Pectineus m., Adductor longus m., Adductor brevis m. Gracilis m.) and posterior divisions (Obturator externus m., Adductor magnus m.). At the medial border, a safe zone can be identified in which the cable wire can be passed with limited risk.

A cadaver study was conducted to understand the relationship of the trans-obturator cable to surrounding structures (Figs. 6 and 7). The obturator foramen is closed by a fibrous membrane. A cable can be passed through the weak medial part of the membrane. The distance between the cable and the obturator canal was 3.0–3.5 cm. Interference with the spermatic cord/round ligament or the femoral vessels should be considered^24^.

## Discussion

Disruption of the pubic symphysis of more than 2.5 cm (1.8–4.5 cm) is a severe injury for which many orthopedic trauma surgeons would agree that operative stabilization is the preferred treatment^25^. In case of minor diastasis, conservative treatment is an option but was abandoned in severe cases due to disadvantages caused by longtime immobilization^26^. Wiring of the pubic symphysis was performed in the past^27^ but the available clinical data is limited, outdated, and only consists of small case series^10,13,28^. Beside the above mentioned clinical reports different biomechanical studies explore the use of a simple wiring technique: Tile et al. fixed the pubic symphysis in one group with double plating (4.5-mm plates), in the second group with four trans-osseous wire loops, and in the third group with absorbable suture material. Wiring resulted in significantly less symphyseal motion than the other methods and they concluded that symphyseal wiring can oppose the tensile load caused during bilateral stance loading at the symphysis^12^. Hofmann compared pubic reconstruction by plate to wiring using cadavers (N = 83). While plate stabilization was stiffer; he mentioned no other difference compared with the wiring technique^11^. Meißner et al. performed a biomechanical study that compared wiring and plating in a cadaver model (N = 24) under physiological conditions. Cut-out of the wires was seen in many specimens and highest stability was provided by plating of the pubic symphysis, what raises concerns about stability of the wiring technique^14^. This are some of the reasons why treatment ultimately evolved to plate fixation over the years^5,29,30^. Unfortunately plating is also a procedure which carries a significant risk for complications. Most frequently subclinical implant loosening (31–80%) is noted in follow-up X-rays^9,31–34^. In case of complete failure or reoccurrence of the diastasis (> 10 mm), revision surgery is required (3–21%)^9,32–34^. Because clinical and biomechanical studies just focus on simple symphyseal wiring, the role of modern cable-systems for this purpose was unclear. Lenz et al. confirmed a superior stability of cable-systems over wiring when fixing long bones. In their biomechanical study a double loop cable cerclage, as used in our group B, has a load-to-failure of mean 2734 N (± 330 N) compared with a single loop wire that only bears 606 N (± 109 N)^16^. Our study confirms high stability of double loop cable fixation of the pubic symphysis; however, we did not compare it to simple wiring.

While passing a cable through the obturator foramen, knowing the location of the obturator canal is important for safety. The obturator canal is a pathway through the obturator membrane containing an artery, vein, and nerve and can be found at the cranio-lateral margin. The obturator artery originates in most cases of the internal iliac vessel (62%)^35^, sometimes forming a connection to the external iliac vessel system, called corona mortis^36^. After passing the obturator canal, the obturator artery divides into an medial and lateral branch to form a vascular circle^37^ or splints into small tributaries without forming such circle^38^. The obturator nerve runs from the obturator canal inferomedially between the adductor longus and brevis muscles and divides into an anterior and posterior division that mainly innervates adductor muscles^37^. Jo et al. conducted a morphometric study of the nerve exit zone in the obturator foramen and showed a median distance to the pubic tubercle of 30 mm^39^. This makes it possible to identify a safe zone (Fig. 7). Trans-obturatorial surgical procedures in the safe zone are also known from other disciplines like vascular surgery (bypass)^40^, general surgery (obturator hernia repair)^37^ and gynecology (midurethral sling procedure)^41,42^. Nevertheless, an overall risk of trans-obturator cable fixation is damage to the bladder, which may later be difficult to treat. Therefore, our possible minimally invasive approach for trans-obturator cable fixation should be carefully investigated. Currently, cranial visualization of the pubic rim and retraction of the bladder remains the favored approach for safety reasons. Beside plating or trans-obturatorial cable fixation other innovative stabilization techniques for the pubic symphysis were described in the past^43–45^. Chen et al. published an article about 45 patients with pubic disruption, treated using percutaneous screw fixation. The authors recommend this technique because of lower blood loss and a better functional outcome^46^. The technique was confirmed by biomechanical findings^47^. Feng et al. introduced percutaneous fixation using a TightRope in combination with an external fixator^48^ and Chen et al. clinically assessed 21 APC-II injuries treated with minimal invasive Endobutton fixation of the pubic symphysis, both without recurrence of the diastasis^49^. Stuby et al. tested a custom made fixation device for flexible fixation and proofed superior stability^50^. In 2020 Fritz et al. evaluated symphyseal fixation by polyaxial pedicle screws and revealed superior strength compared to plating^51^. Currently, none of these alternative fixation techniques is in widespread use but it demonstrates the continues quest for a new technique.

## Limitations

**Figure 8:**
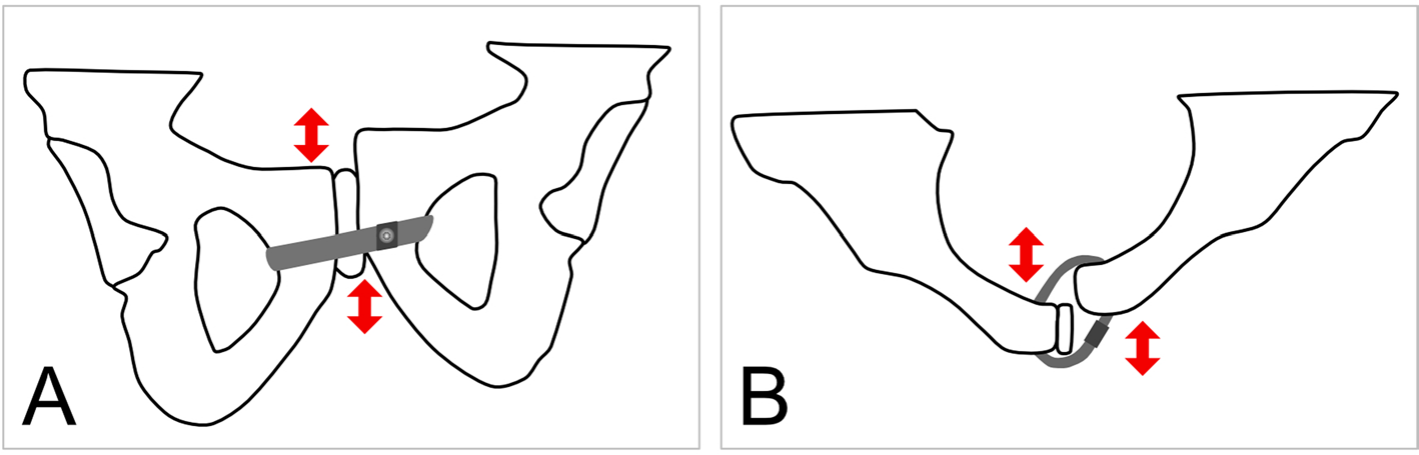
Complication that may occur using trans-obturator cable fixation. (**A**) Horizontal dislocation of the pubic rim is possible because of shear stress. (**B**) In case of a very unstable posterior pelvic ring that has not been stabilized, dislocation of the pubic rim in the axial plane may be a problem.

The use of synthetic pelvic models instead of cadaver specimens is controversial in orthopaedic research but there are some reasons why synthetic bones are increasingly used. They are affordable at a justifiable budget, have similar biomechanical properties than cadaver specimens, are easily available without logistical problems, have almost no variability between specimens and are ethically uncritical^52^. In contrast, there is a high variability in biomechanical properties between cadaver specimens and the specimens themselves represent disproportionally the elderly population^53^. Therefore, cadaver specimens often do not adequately simulate the biomechanical behavior of orthopaedic implants. Especially polyurethane foam based bone models were intensely tested and confirmed for biomechanical studies^54^. Still, the synthetic pelvic model used here has no ligaments or muscular attachments. The load vectors are therefore expected to differ in cadaver models. In summary, a synthetic pelvis model is useful for a comprehensive statistically preliminary testing but cadaver studies should be considered before first-in-human study. Further, the monoaxial, single leg load applied in the biomechanical testing does not simulate bilateral strain. No implant loosening was observed in the plating group what may be a result of the low number of test cycles, despite a high number used in our pretests. Moreover, loosening of the cable fixation was not detected, making it difficult to verify the cable to be a more dynamic fixation. Placing the cerclage in the desired position could be more difficult in real surgery than it is in a synthetic pelvic model. The Pfannenstiel approach only allows for easy dissecting of the cranial corner of the foramen through which to pass the wire. Further studies are required to assess the ideal surgical approach^55^. Another concern is loosening of the cable-system leading to displacement of pubic bones in the axial and sagittal planes caused by continuous movement in the vertical and horizontal axis of the pelvis (Fig. 8). Although more stable results may be achieved using pre-bent 6-hole plates with options for dynamic compression and screw locking mechanisms, we chose a 4-hole symphyseal non-locking plate for our control group^56^. Finally, the unilateral load applied in our single leg stance model can result in vertical translation and compression of the pubic symphysis, but is unlikely to cause re-widening. The focus of upcoming biomechanical studies should include test settings able to provoke a re-diastasis of the pubic symphysis. A two-leg alternating load model may be appropriate^21^.

## Conclusion

Our study confirms adequate stability of symphyseal plating and demonstrates similar biomechanical properties of trans-obturator cable fixation. Passing a cable at the medial margin of obturator foramen seems possible and relatively safe.

## Data Availability

The datasets generated during and/or analyzed during the current study are available from the corresponding author on reasonable request.
